# Novel thoracoscopic segmentectomy combining preoperative three-dimensional image simulation and intravenous administration of indocyanine green

**DOI:** 10.1093/icvts/ivac064

**Published:** 2022-03-03

**Authors:** Natsumi Matsuura, Hitoshi Igai, Fumi Ohsawa, Kazuki Numajiri, Mitsuhiro Kamiyoshihara

**Affiliations:** Department of General Thoracic Surgery, Japanese Red Cross Maebashi Hospital, Maebashi, Japan

**Keywords:** Segmentectomy, Three-dimensional computed tomography, Indocyanine green, Surgical margin

## Abstract

**OBJECTIVES:**

The aim of this study is to assess prospectively the validity and feasibility of segmentectomy using preoperative simulation and intravenous indocyanine green (ICG) with near-infrared (NIR) light thoracoscope to ensure a sufficient surgical margin.

**METHODS:**

This study was a prospective, single-centre, phase II, feasibility study. From February to July 2021, 20 patients were enrolled in this study. All patients underwent preoperative three-dimensional computed tomography angiography and bronchography using simulation software. The dominant pulmonary artery of the targeted segment was selected to determine the dissection line and measure the surgical margin to the tumour. Intraoperatively, after the planned dissection of the pulmonary artery, ICG (0.3 mg/kg) was administered intravenously and observed with NIR, and dissection was performed along the line determined by preoperative simulation. Postoperatively, the pathological margin was compared with the simulation margin.

**RESULTS:**

All surgeries were performed via an uniport (3.5–4.0-cm skin incision). The regions of segmentectomy were S2, S3, S6, S9 + 10 and S10 of the right side and S1 + 2 + 3, S3, S3 + 4 + 5, S6 and S8 of the left side. The difference between the simulation margin and the pathological margin was not significant (simulation 30.5 ± 10.1 vs pathological 31.0 ± 11.0 mm, *P* = 0.801). The simulation margin was well correlated with the pathological margin (*R*^2^ = 0.677). The proportion of cases successfully achieving the pathological margin of error of plus or minus 10 mm of the simulation margin was 90% (18 of 20 cases).

**CONCLUSIONS:**

The combination of preoperative three-dimensional computed tomography simulation and ICG–NIR was effective for securing a sufficient margin in segmentectomy.

**Figure:**
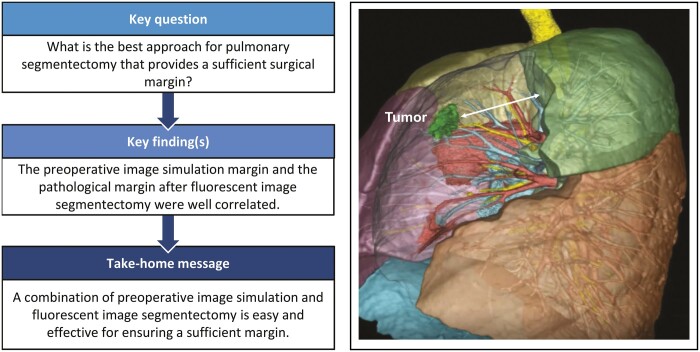

## INTRODUCTION

Although the standard radical surgery for primary lung cancer is still lobectomy [1], an early-stage non-small-cell lung cancer with ground-glass opacity (GGO) on thin-section computed tomography (CT) has been shown to have a very good prognosis, and segmentectomy has become widespread [2–5]. Segmentectomy is also an effective surgical procedure for frail cases that cannot tolerate radical surgery for primary lung cancer and cases with metastatic tumours near the hilum. When performing segmentectomy for a malignant tumour, ensuring a sufficient margin around the tumour, whether intentionally or passively, is the most important aspect. However, it is sometimes difficult to confirm that a sufficient surgical margin has been secured intraoperatively when the tumour is impalpable because it is small or GGO dominated.

In 2009, a novel intersegment identification method was reported in which intravenous indocyanine green (ICG) and near-infrared (NIR) light thoracoscope were used in combination [6, 7]. This is a method for identifying the intersegmental plane that uses the properties of ICG to visualize blood flow on the surface of the lungs, and several clinical studies of its use have been reported [8–10]. However, the most important issue in segmentectomy is to identify the anatomically correct intersegmental plane and to ensure a sufficient surgical margin from the tumour, but there are no reports of a prospective evaluation of the association between intersegmental identification using ICG and the surgical margin.

We recently introduced the three-dimensional (3D) analyzer ‘Ziostation2’ (Ziosoft, Inc., Tokyo, Japan), which enables us to simulate preoperatively the relationship between tumours and intersegmental planes visualized by intravenous administration of ICG. This time, the validity and feasibility of segmentectomy using this preoperative simulation and ICG-NIR to ensure a sufficient surgical margin were assessed prospectively, examining the discrepancy between the simulated margin and the actual pathological margin.

## PATIENTS AND METHODS

### Ethical statement

This study was registered in the Japan Registry of Clinical Trials on 1 February 2021 with the registration No. jRCT1030200352 and was approved by the institutional ethics board of Maebashi Red Cross Hospital (No. 2020-03).

### Patient enrollment

This was a prospective, single-centre, phase II, feasibility study. Written informed consent was obtained from all enrolled patients. The inclusion criteria included: (i) suspected primary lung cancer in which CT demonstrates a pure or part-solid ground-glass nodule with solid size of 1 cm or less confirmed as clinical stage 0-IA1 (Tis-1aN0M0) according to the 8th edition of the TNM classification by careful preoperative staging with CT and/or PET–CT (i.e. intentional limited surgery); (ii) metastatic lung tumour located near the hilum and unable to undergo wedge resection; and (iii) suspected or diagnosed clinical N0M0 primary lung cancer in a patient who cannot tolerate radical surgery due to complications and poor pulmonary function (i.e. passive limited surgery). The exclusion criteria included: (i) a history of allergy to iodine-containing contrast agents and (ii) women during pregnancy or breastfeeding. The sample size of 20 was justified by rationale about feasibility, precision about the mean and variance and regulatory considerations [11].

### Preoperative simulation

**Figure 1:**
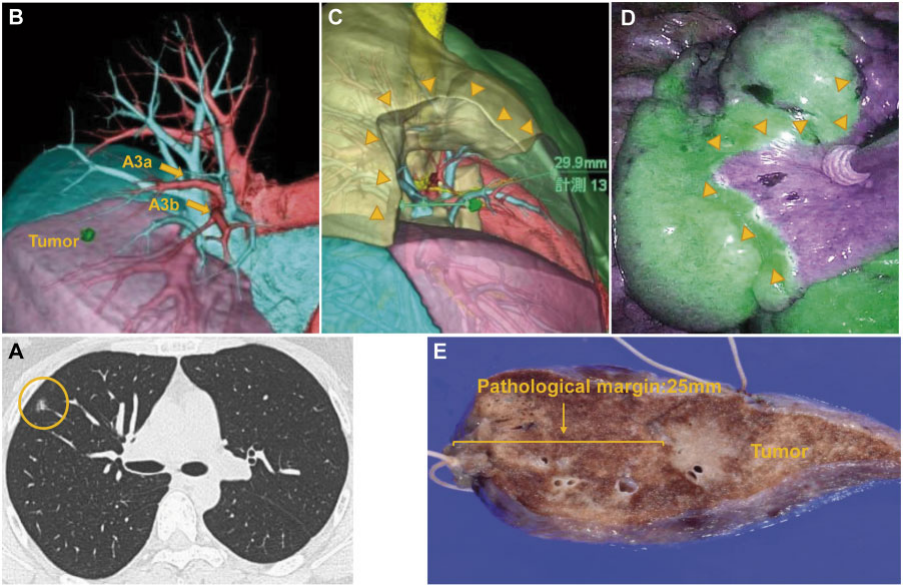
A case of a small-sized part-solid ground glass opacity located at right S3a (**A**). Three-dimensional computed tomography angiography showing the dominant pulmonary arteries (A3a and A3b) and the location of the tumour (**B**). Based on the perfusion area of A3a and A3b, simulation of right S3 segmentectomy shows that the margin from the intersegmental line to the tumour is 29.9 mm (**C**). Intraoperative indocyanine green image using a near-infrared light thoracoscope shows clear visualization as simulated (**D**). A resected lung specimen. The pathological margin in this case is 25 mm (**E**). CT: computed tomography.

Contrast-enhanced, high-resolution CT (thickness ≤1 mm) was performed for all enrolled patients prior to surgery. These data were transferred to a workstation ‘Ziostation2’ (Ziosoft, Inc., Tokyo, Japan) and 3D-CT angiography and bronchography were created automatically. In this software, tumour location was automatically or manually indicated. In the simulation workstation, the segment that each pulmonary artery dominated was shown as non-coloured area when we plotted a branch of pulmonary artery which was planned to be resected in the operation while other segment was coloured. The boundary line between coloured and non-coloured area, considered as ‘virtual intersegmental plane’, was expected to correspond to one which would be identified via infrared thoracoscopy with administration of ICG in the operation. Finally, the surgical margin between the tumour and the closest boundary line was calculated (Fig. 1). When the surgical margin was insufficient (<20 mm) and the tumour was near the hilum, shifting the staple line was difficult and dangerous due to possible vessel injury; therefore, we extended the planned resected area in the simulation by choosing additional branches of pulmonary artery. Otherwise, we planned to extend the resected area by shifting the staple line towards distant direction from the target tumour in the actual operation. We set the distance to shift the staple line, and their sum is used as the simulation margin. Therefore, the simulation margin was always set over 20 mm.

### Surgical procedure and evaluation of indocyanine green demarcation

All patients were operated under general anaesthesia in the lateral decubitus position with differential lung ventilation. The operator always stood on the ventral side and the assistant on the dorsal side of the patient. A 3.5–4.0-cm skin incision was made in the fourth intercostal anterior axillary line for right upper lobe segmentectomy or the 5th intercostal anterior axillary line for other types of segmentectomy and an XS Alexis wound retractor (Applied Medical, Rancho Santa Margarita, CA, USA) was fitted. A 10-mm 30° thoracoscope (VISERA ELITE II OLYMPUS CLV-S200-IR, Olympus Deutschland GmbH, Hamburg, Germany) was immobilized on the dorsal side of the wound margin, with the ventral side providing space for the operator to manipulate the scope. Vessel and bronchial transection were in principle carried out with an automated suturing device, but suture ligation with 3–0 silk was performed if required by the vessel diameter. In our criteria, intentional limited surgery is performed only for a pure or part-solid ground-glass nodule with solid size of 1 cm or less confirmed as clinical stage 0-IA1 (Tis-1aN0M0). Therefore, we usually did not perform lymphadenectomy in these cases. In some cases, we sampled hilar lymph nodes.

**Figure 2:**
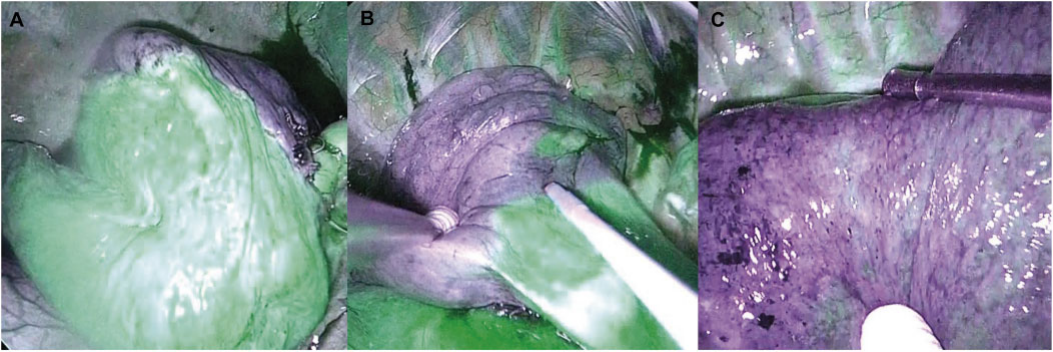
The indocyanine green demarcation score. Clear visualization without interruption (3 points, excellent) (**A**). Partially unclear but generally identifiable (2 points, fair) (computed tomography). Difficult to identify (1 point, poor) (**C**).

After division of all the dominant vessels and bronchi that were planned in the simulation, an intravenous bolus of ICG (0.3 mg/kg/10 ml) was administered at an administration rate of 300 ml/h [12], and observation was performed with NIR. The segments to be resected were not stained, so the boundary was marked by electrocautery. At the same time, the intersegmental demarcation was assessed by 2 authors (Natsumi Matsuura and Hitoshi Igai) on ICG–NIR by scoring for each plane: clear visualization without interruption (3 points, excellent); partially unclear but generally identifiable (2 points, fair); and difficult to identify (1 point, poor) (Fig. 2). The number of intersegmental planes for each segmentectomy is shown in Table 1. After marking the ICG demarcation line, all intersegmental planes were divided by staplers.

**Table 1:** The number of intersegmental planes for each segmentectomy

Segmentectomy	Intersegmental plane	Number	Total number
S2	Between S2 and S1	2	4
	Between S2 and S3	2	
S3	Between S3 and S1	2	4
	Between S3 and S2	2	
S6	Between S6 and basal segment	2	2
S8	Between S8 and S9	2	2
S9 + 10	Between S9 + 10 and S6	2	4
	Between S9 and S8	2	
S10	Between S10 and S6	2	4
	Between S10 and S9	2	
Left S1 + 2 + 3	Between S3 and S4	2	2
Left S3 + 4 + 5	Between S3 and S1 + 2	2	2

### Postoperative management

The chest drainage tube was removed after confirming that there was no air leakage and the daily drainage amount was <300 ml. The patients could be discharged if the chest X-ray taken the day after chest drain removal did not show any problems.

### Measurement of the surgical margin

To obtain inflated-fixed lung specimens, 10% formalin was injected through the bronchial stumps and clamped when the lungs were fully inflated. Then, entire specimens were immersed in the same formalin for about 24 h. The surgical specimens were examined and diagnosed by a pathologist. A pathologist also measured the shortest distance from the resection line to the tumour. In difficult cases, Natsumi Matsuura helped a pathologist to calculate the surgical margin while checking the simulation image.

### Statistical analysis

The primary outcome of this study was the proportion of cases who achieved the pathological margin of error of plus or minus 10 mm of the simulation margin. Because we set the simulation margin over 20 mm as mentioned above, we consider that an error of 10 mm or less would be acceptable. Secondary end points included the score of segmental line demarcation.

Wilcoxon signed rank-sum test was used to compare the difference between the simulation margin and the pathological margin. The Mann–Whitney *U*-test was used to compare the ICG demarcation score between subgroups. Results were considered significant for values of *P *<* *0.05. The Pearson product-moment correlation coefficient was used to calculate the correlation coefficient of the simulation margin and the pathological margin. All statistical analyses were performed with EZR (Saitama Medical Center, Jichi Medical University, Saitama, Japan), which is a graphical user interface for R (The R Foundation for Statistical Computing, Vienna, Austria). More precisely, it is a modified version of R commander designed to add statistical functions frequently used in biostatistics.

## RESULTS

### Patients’ characteristics

Twenty patients were enrolled in this study between February 2021 and July 2021. The characteristics of all patients [5 males, 25.0%; 15 females, 75.0%; mean age 64.9 (range, 29–84) years] are shown in Table 2. The regions of segmentectomy were S2, S3, S6, S9 + 10 and S10 of the right side and S1 + 2 + 3, S3, S3 + 4 + 5, S6 and S8 of the left side, including 15 intentional limited resections. The indications for passive limited resection for primary lung cancer included interstitial pneumonia in 1 case and post-lobectomy of the contralateral side in 1 case.

**Table 2:** Characteristics and surgical outcomes

Variables	Frequency (*n* = 20)
Age, years	64.9 ± 12.2
Sex	
Female	15 (75.0)
Male	5 (25.0)
Preoperative respiratory function test	
FEV1(l)	2.29 ± 0.58
FEV1%	75.5 ± 7.6
%FEV1	102.4 ± 17.9
Tumour diameter, mm	13.7 ± 6.2 (7–29)
Consolidation diameter, mm	7.0 ± 6.3 (0–25)
Segmentectomy regions	
Right	10 (50)
S2	4 (20)
S3	3 (15)
S6	1 (5)
S9 + 10	1 (5)
S10	1 (5)
Left	10 (50)
S3	1 (5)
S1 + 2 + 3	4 (20)
S3 + 4 + 5	1 (5)
S6	2 (10)
S8	2 (10)
Reason for limited resection	
Intentional	15 (75)
Passive	2 (10)
Others (metastatic tumour)	3 (15)
Operation time. min	129 ± 34 (85–230)
Blood loss, ml	15 ± 21 (0–50)
Postoperative drainage, days	1.1 ± 0.3 (1–2)
Postoperative hospitalization, days	2.3 ± 0.4 (2–3)
Postoperative complications	1 (5)
Delayed pneumothorax	1 (5)

Data are shown as mean ± standard deviation (range) or number (%). FEV1: forced expiratory volume in one second; PET: positron emission tomography.

On preoperative pulmonary function testing in all patients, forced expiratory volume in 1 s was 2.29 ± 0.58 l (102.4 ± 17.9%, predicted) and forced expiratory volume in 1 s/forced vital capacity was 75.5 ± 7.6%. The mean size of the tumour was 13. 7 ± 6.2 (range 7–29) mm, and consolidation size was 7.0 ± 6.3 (range 0–25) mm.

### Surgical outcomes

Surgical outcomes are shown in Table 2. The mean operation time was 129 ± 34 min with the mean bleeding of 15 ± 21 ml. Postoperative drainage lasted 1.1 ± 0.3 days and the duration of postoperative hospitalization was 2.3 ± 0.4 days.

Postoperative complications occurred in only 1 patient (5.0%). The patient who had left S3 segmentectomy and lower lobectomy for a simultaneous ipsilateral lung cancer had delayed pneumothorax. There were no ICG-related adverse events.

### Indocyanine green demarcation score

**Figure 3:**
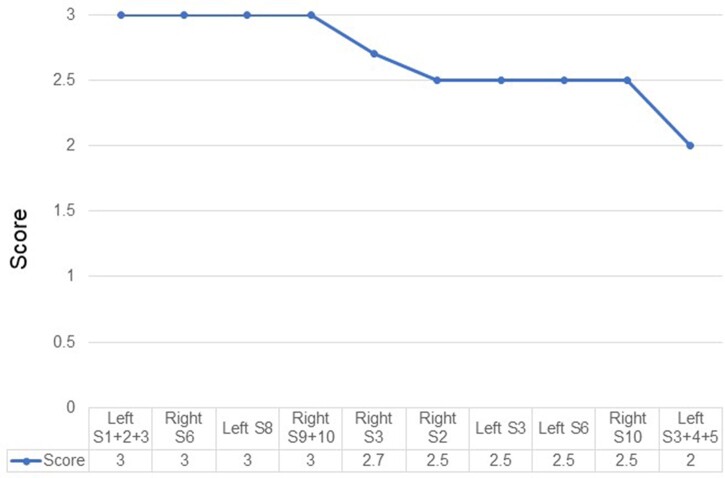
The intersegmental demarcation score. The method to calculate the score is to divide the total score by the number of intersegmental planes (S2/3/8/9/9 + 10: 4, S6/S1 + 2 + 3/S3 + 4 + 5: 2). ICG: indocyanine green.

It was possible to identify 59 of 64 intersegmental planes in total, for a success rate of 92.2%. The result of the ICG demarcation score of each segment is shown in Fig. 3. The method to calculate the score was to divide the total score by the number of intersegmental planes (Table 1). There was no significant difference between simple and complex segmentectomies (simple 2.8 ± 0.3 vs complex 2.6 ± 0.3, *P* = 0.34). There was also no significant difference between upper and lower lobe segmentectomies (upper lobe 2.5 ± 0.4 vs lower lobe 2.8 ± 0.3, *P* = 0.24).

### Comparison of simulation margin and pathological margin

**Figure 4:**
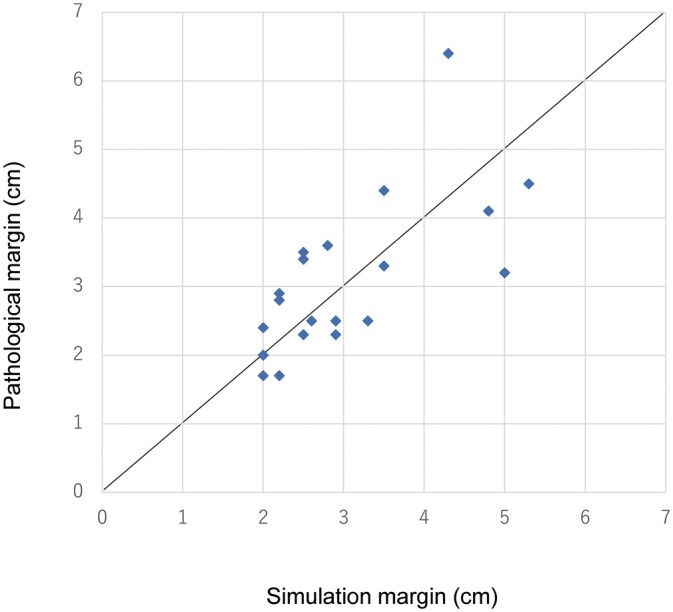
The relationship between the simulation margin and the pathological margin.

Pathological results are shown in Table 3. Surgical margins for malignancy were negative in all patients (100%). The difference between the simulation margin and the pathological margin was not significant (simulation 30.5 ± 10.1 vs pathological 31.0 ± 11.0 mm, *P* = 0.647). The simulation margin was well correlated with the pathological margin (*R*^2^ = 0.671, *P* = 0.00168, Fig. 4). The proportion of cases that successfully achieved the pathological margin of error of plus or minus 10 mm of the simulation margin was 90% (18 of 20 cases).

**Table 3:** Pathological results

Variables	Frequency (*n* = 20)
Pathological diagnosis	
Primary lung cancer	15 (75)
Adenocarcinoma	13 (65)
pTisN0M0	7 (35)
pT1miN0M0	4 (20)
pT1aN0M0	1 (5)
pT1bN0M0	1 (5)
pT1cN0M0	1 (5)
pT2aN0M0	1 (5)
Squamous cell carcinoma	
pT1bN0M0	1 (5)
Small cell carcinoma	
pT2aN0M0	1 (5)
Metastatic lung tumour	3 (15)
Benign tumour (hamartoma)	2 (10)
	
Surgical margin	*P *=* *0.801
Simulation margin, mm	30.5 ± 10.1 (20–53)
Pathological margin, mm	31.0 ± 11.0 (20–64)

Data are shown as mean ± standard deviation (range) or number (%).

## DISCUSSION

In this study, there was a slight discrepancy between the simulation margin and the pathological margin, and a sufficient surgical margin was achieved in all cases. Although the use of ICG-NIR in segmentectomy has been widely reported, most such reports have focused on whether the intersegmental plane is clearly visualized, and to the best of our knowledge, there has been no previous report of a prospective study of the key factor of the tumour margin. This is the first prospective report of a procedure including the determination of the resection line for segmentectomy on preoperative 3D-CT with reference to the pulmonary artery and simulation of the margin, the performance of segmentectomy using both intravenous ICG administration and NIR thoracoscopy and confirmation of consistency with the pathological margin.

Various methods of identifying the intersegmental plane have been reported, including inflation-deflation, jet ventilation, virtual-assisted lung mapping (VAL-MAP) and intravenous or transbronchial ICG administration [13–20], but the best method remains controversial. The classical method is inflation–deflation, which has the advantages of simplicity and of not requiring any special instrumentation, but, in thoracoscopic surgery, the inflated lung may obstruct the operating field, particularly if it is emphysematous, and a comparatively long time is required to wait for the lung to deflate. Jet ventilation, in which the segment to be resected is selectively inflated, was reported by Okada *et al.* [13]; it enables different segments to be rapidly and easily identified by inflating only the segment to be resected, but it requires the participation of an anesthesiologist and familiarity with the procedure. Transbronchial ICG administration has a similarly high reported success rate, but in addition to the proficiency required to perform bronchoscopy procedures and the danger of splashing neighbouring segments, the fact that this is an off-label procedure is also an issue [16, 17].

Intravenous ICG administration and NIR thoracoscopy are now widely used for identification of the intersegmental plane [18–20]. Because segment identification by ICG does not require the lung to be inflated, it does not impede the operator’s field of view in thoracoscopic surgery. Intravenous ICG administration is simple and quick to perform, with only a small amount used each time, and repeated administration is therefore feasible if required. It also does not involve the additional radiation exposure of VAL-MAP and intraoperative CT. Its disadvantages include the fact that it cannot be used for patients allergic to iodine contrast agents and that visualization is only maintained for a short time, although this is sufficient for the segment to be marked with electrocautery or a similar method.

Our method is extremely useful when the tumour is located near the intersegmental plane and not palpable intraoperatively because it is small or GGO dominated. In cases other than the above, it is generally easy to ensure a sufficient margin in segmentectomy even in thoracoscopic surgery. Sato *et al.* [21] reported VAL-MAP-assisted segmentectomy, which enables surgeons to determine oncologically appropriate resection lines. VAL-MAP is a novel technique that allows for bronchoscopic multi-spot dye markings to provide ‘geometric information’ on the lung surface, using 3D virtual images [14, 15]. However, it requires preoperative bronchoscopy and additional instruments, and in addition to the complexity of the procedure and the need for familiarity on the part of the operator, its cost is also a problem. Wu *et al.* [22] reported combined subsegmentectomy for achieving a sufficient surgical margin for an intersegmental nodule. Combined subsegmentectomy can ensure that a safe margin is achieved by placing the intersegmental nodules in the central area of the involved adjacent subsegments. However, it requires complicated procedures to dissect and transect the subsegmental vessels and bronchi, and the average operation time was relatively long (190 ± 54.9 min). In this system, we freely choose pulmonary artery branches which will be divided in the operation, which can modify the extent of resected segment. Therefore, we can manage the surgical margin on the image by choosing additional pulmonary artery branch even when the surgical margin is insufficient. Otherwise, we can manage the surgical margin by shifting the staple line towards distant direction from the target tumour. Comparing VAL-MAP or combined subsegmentectomy, this method is quite simple and easy to perform. It is effective even for intraoperatively impalpable tumours, providing a secure margin for tumour resection.

In the present study, we found good correspondence between the ‘virtual’ and ‘actual’ intersegmental plane, which enabled us to perform appropriate thoracoscopic segmentectomy with sufficient surgical margin. Moreover, we used ICG demarcation score in each intersegmental plane to evaluate identification of the intersegmental plane in the operation quantitatively. In this series, intersegmental identification success rate was 92.2% and this result was comparable to previously reported rates, which ranged from 84.6∼95% [8, 23, 24]. Three patients in this series had unconvincing results due to severe interstitial pneumonia in 1 case and emphysema in 2. There have been few reports verifying the difference of ICG demarcation depending on the type of segmentectomy, we found that there were no significant differences between simple and complex segmentectomies and between upper lobe and lower lobe segmentectomies.

Intersegmental resection methods can be broadly divided into the use of electrocautery and the use of a stapler [25, 26]. The use of electrocautery to create the intersegmental plane reportedly increases the risk of a postoperative pulmonary fistula [25], and in the present study, a stapler was used for all intersegmental transections. In complex segmentectomy by uniportal VATS, the angle of insertion of the stapler is restricted to a single direction, but it was possible to carry out without problems by moving the lung significantly and matching it to the stapler insertion angle. Intravenous ICG administration is a method of creating an intersegmental plane with reference to a line visualized on the surface of the lung, and using a stapler for resection is very reasonable.

### Limitations

There are several limitations in this study. This was a small sized, single-centre, non-comparative study. Moreover, the study period was too short to evaluate long-term outcomes. In particular, it is necessary to examine the long-term oncologic outcomes of cases of primary lung cancer. In addition, ICG is sometimes not clearly visualized in the presence of interstitial pneumonia or severe emphysema [8, 9]. If there is a poorly visualized area, the intersegmental planes have to been divided while combining other methods such as the inflation–deflation technique and confirming the location of the tumour to ensure a sufficient margin.

## CONCLUSION

In conclusion, uniportal thoracoscopic segmentectomy was conducted in 20 patients by combining a preoperative 3D-CT simulation based on the pulmonary artery with intravenous ICG administration, and the tumour was successfully removed with a sufficient margin in all cases. This method provides a sufficient tumour margin, which is the most important factor in segmentectomy, and it can be conducted safely and easily.

## ABBREVIATIONS

3D: Three-dimensional
CT: Computed tomography
GGO: Ground-glass opacity
ICG: Indocyanine green
NIR: Near-infrared
VAL-MAP: Virtual-assisted lung mapping
